# Successful bilateral electroconvulsive therapy for catatonia presenting with novel climbing behavior in an adolescent with *CACNA1A* pathogenic variant and autism spectrum disorder: a case report

**DOI:** 10.3389/fpsyt.2026.1782341

**Published:** 2026-04-10

**Authors:** Lana Abdole, Hannah Louise Reynard, H. Yavuz Ince, Alexander Palffy, James Jackson, Neera Ghaziuddin

**Affiliations:** Child and Adolescent Psychiatry, University of Michigan, Ann Arbor, MI, United States

**Keywords:** autism spectrum disorder, bilateral electroconvulsive therapy, *CACNA1A* pathogenic variant, catatonia, climbing behaviors, ECT

## Abstract

Pathogenic gene variants are relatively common in patients with neurodevelopment disorders comorbid with catatonia. In this report, we describe diagnosis and the treatment of catatonia in a 16-year-old boy with a CACNA1A pathogenic variant (which is associated with deficit in neuronal communication and neurotransmitter release). This case describes an adolescent with a CACNA1A pathogenic variant and autism spectrum disorder who develops catatonia and was safely and successfully treated with bilateral electroconvulsive therapy (ECT). Additionally, we describe a unique presenting symptom of climbing behaviors that was contextualized as a symptom of catatonia and discuss psychopharmacological interventions. To monitor treatment response, we utilized observational data collected by a behavior analyst, as well as physician and parent report via Busch-Francis Catatonia Rating Scale and Catatonia Impact Scale respectively. All three reports showed significant improvement in catatonic symptoms following treatment with ECT. The overall aim is to improve the management of catatonia in rare genetic disorders, demonstrate effective use of ECT in cases with this pathogenic variant, and provide guidance to clinicians and hope for patients and families struggling with this comorbidity.

## Introduction

*CACNA1A* variants are associated with multiple neurological disorders, and clinically, pathogenic variants span a spectrum of clinical phenotypes (2). Additionally, variations in *CACNA1A* genes were found to be associated with neurodevelopmental changes, including autism spectrum disorder (ASD), intellectual disability, developmental delay, and other neurodevelopmental disorders (3). Although there are a few reports specifically regarding *CACNA1A* disorder and the risk for catatonia, the literature provides support that those with gene mutations and neurodevelopmental delays may be at a greater risk for catatonia (1, 4). A literature search revealed a single case report regarding an adolescent with a pathogenic *CACNA1A* variant who had presented with catatonia and was treated with unilateral electroconvulsive therapy (ECT) (5). In this case, prolonged neurologic deficits and MRI changes occurred following ECT. The authors hypothesized that the MRI changes and acute decompensation were potentially related to their underlying *CACNA1A* disorder, while the ECT may have precipitated additional complications. Thus, the authors recommended caution when considering ECT in this patient group (5).

In the current paper, we present a case involving an adolescent with a *CACNA1A* pathogenic variant, ASD and attention-deficit/hyperactivity disorder (ADHD) who presented for a second opinion for the treatment of neuropsychiatric symptoms consistent with catatonia. Past treatment history revealed minimal response to pharmacology, which is discussed in this case presentation. The patient was hospitalized to a specialized inpatient child psychiatry unit for work-up and for consideration for the initiation of ECT. Clinical consensus was reached to use bilateral ECT for the treatment of catatonia, which was well tolerated and showed positive improvement with improvement evidenced early in the course of ECT course. The improvement was noted both by the clinicians and was corroborated by parental reports.

Here, we discuss patient’s unique presentation, treatment management, and efficacy of ECT in a patient with a *CACNA1A* pathogenic variant.

## Case presentation

This is a 16-year-old male with a past psychiatric history of ASD, *CACNA1A* pathogenic variant (specific variant c.3414delC, p.Lys1138Argfs*48), intellectual disability, and ADHD who was referred to our outpatient clinic for ongoing management of catatonia. During the previous year prior to his presentation, the patient had experienced a significant decline in functioning. At baseline, the patient had stereotypic behaviors in the context of ASD (such as tapping and rocking/rolling back and forth), was continent during daytime, and able to engage in family activities (such as visiting restaurants, attend church, travel, and participate in outdoor activities). Symptoms observed over the past year included bowel and bladder incontinence, increased hyperactivity, increased frequency and intensity of stereotyped behaviors, increased impulsivity (which included eloping from school and home), and aggression (grabbing and pushing). The family noticed negativism, withdrawal, and ambitendency. Weight loss and insomnia throughout the night were also observed. These new symptoms were concerning for catatonia. Though incontinence is not listed as a catatonic symptom in the Diagnostic and Statistical Manual of Mental Disorders, 5^th^ edition (DSM-5), regression of skills, such as incontinence, has been observed in cases of catatonia (6). As medical work up was negative for any etiology for incontinence, it was suspected to be related to his catatonia diagnosis. Furthermore, he developed a novel repetitive behavior which included climbing with no clear purpose (such as climbing trees, buildings, the ceiling in the classroom) leading to safety concerns. As this was a new behavior inconsistent with his baseline behaviors, this was conceptualized as a novel stereotypy and a perseverative behavior in the context of a catatonia diagnosis.

Although the patient was previously admitted inpatient to an outside hospital for eight days where he was first diagnosed with excited catatonia, the treatment had failed to elicit any significant improvement. During the outside hospitalization, he was first started on a benzodiazepine, which is first choice treatment for catatonia. Clonazepam was initiated and titrated to 1 mg three times a day. He was also started on valproic acid 250 mg twice a day. Sleep had improved with clonazepam, however, there was no significant improvement noted in other catatonia symptoms. Concerns for his safety were ongoing due to the climbing behavior in particular. Multiple additional medication trials had also been attempted by his outpatient psychiatrist to target his symptoms.

The patient presented to our center approximately 3 months after discharge from the outside hospital for an outpatient ECT consultation. At the time of his presentation, his parents reported a 30% improvement relative to his ill baseline. Notable findings were Busch-Francis Catatonia Rating Score (BFCRS) = 19 (7), which increased further into the admission to 28 and Clinical Global Impressions Scale (CGI-S) (8) was 6 (severely ill). The Catatonic Impact Score (CIS) completed by the parents showed a total frequency score of 61, a total impact score of 78, and a total of 139. The CIS adheres to the format of the fully validated Autism Impact Measure developed by Kanner and Mazurek and queries symptoms enumerated in the BFCRS. It has been used in other publications to monitor catatonia symptoms (9). Medications at the time were clonazepam 1 mg three times a day, valproic acid 500 mg twice a day, trazodone 100 mg nightly, propranolol 200 mg three times a day, hydroxyzine 50 mg as needed, and melatonin 10 mg nightly. Treatment recommendations at our institution were to gradually optimize the benzodiazepine as tolerated, as higher doses are commonly required for treatment of catatonia (10). He had been on clonazepam 3 mg a day for 3 months. His outpatient physician had briefly attempted to increase the medication by 1 mg; however, family thought his agitation worsened so it was reduced back to prior dose. We recommended trial of adding lorazepam to regimen to optimize benzodiazepine dose. ECT was discussed as a second-line option if there was failure to improve or worsening in symptoms. Propranolol was tapered down due to lack of benefit and ongoing impulsivity despite a high dosage.

Approximately 5 months after the initial outpatient consultation visit, the patient was admitted to our child and adolescent psychiatry neurodevelopmental unit (a tertiary referral center at a mid-west academic center). At start of the hospitalization, patient was on the above-mentioned regimen with the exception that lorazepam had been added with dose of 0.25 mg four times a day for approximately 5 months and valproic acid had been slightly increased. No noticeable change in symptoms had been noted with medication changes. While the work-up for initiating ECT was in progress, the inpatient team consolidated the benzodiazepines to one and attempted to further optimize the dose. Lorazepam was discontinued and clonazepam was increased up to 2 mg three times a day. No significant change was noted in catatonic symptoms. As clonazepam was increased there was concern for worsening disinhibition as patient began disrobing and displaying increased hyperactivity.

After obtaining consensus from three child and adolescent psychiatrists and informed consent from the parents, the laboratory work-up for ECT was completed that included: a Chest X-ray, Electrocardiogram (EKG), Complete Blood Count (CBC), Thyroid Stimulating Hormone (TSH) with reflex, Complete Metabolic Panel (CMP), Ammonia, Pediatric Glomerular Filtration Rate (GFR), Creatinine Kinase (CK), Valproic Acid (VPA) level. Neurology work-up was extensive and in part included a Magnetic Resonance Imaging (MRI) brain, lumbar puncture (LP), Cerebrospinal Fluid (CSF) autoimmune encephalitis panel, additional labs to rule out auto-immune process, and an Electroencephalogram (EEG). Please see supplemental section for full list of labs. All laboratory results failed to identify any medical etiology for catatonia.

ECT treatment was initiated with bilateral electrode placement, using the MECTA device, at three times a weekly schedule. The total electrical charge was gradually increased to maximum dose of 576 mC. During the induction for anesthesia, patient received glycopyrrolate to prevent bradycardia and reduce secretions, ketorolac for pain, ondansetron for nausea, and flumazenil, which competitively binds with benzodiazepine receptors in the central nervous system to improve the quality and the duration of the ECT-induced seizures (11, 12). Caffeine (intravenous) was added on ECT #31 in order to further achieve therapeutic seizure duration (13). During the ECT course, valproic acid was tapered off and discontinued given a lack of efficacy and to optimize seizure duration of ECT. Propranolol was also tapered off, given a lack of efficacy in improving symptoms of impulsivity.

**Figure 1:**
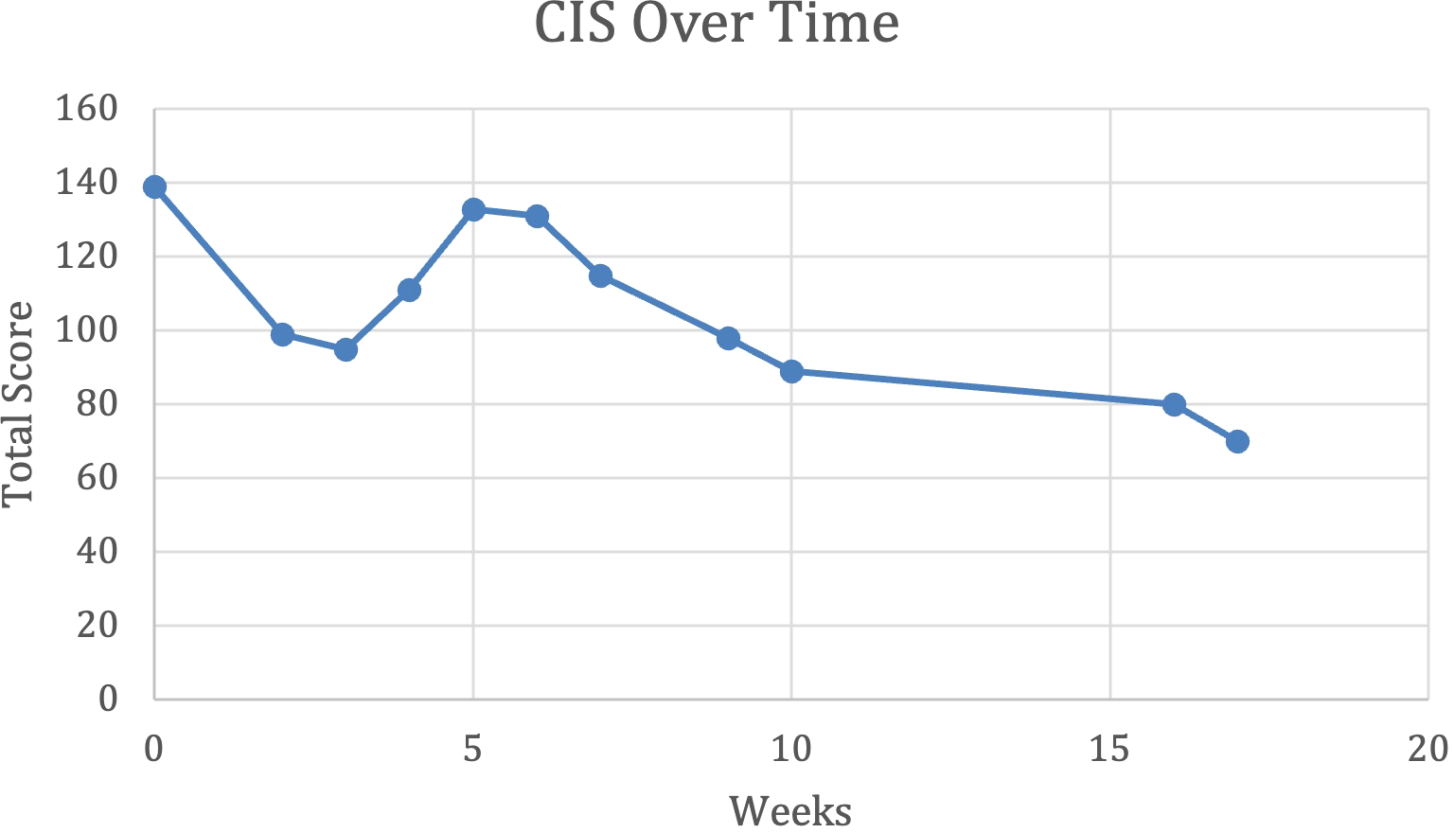
Catatonia impact score (CIS) over time in weeks.

**Figure 2:**
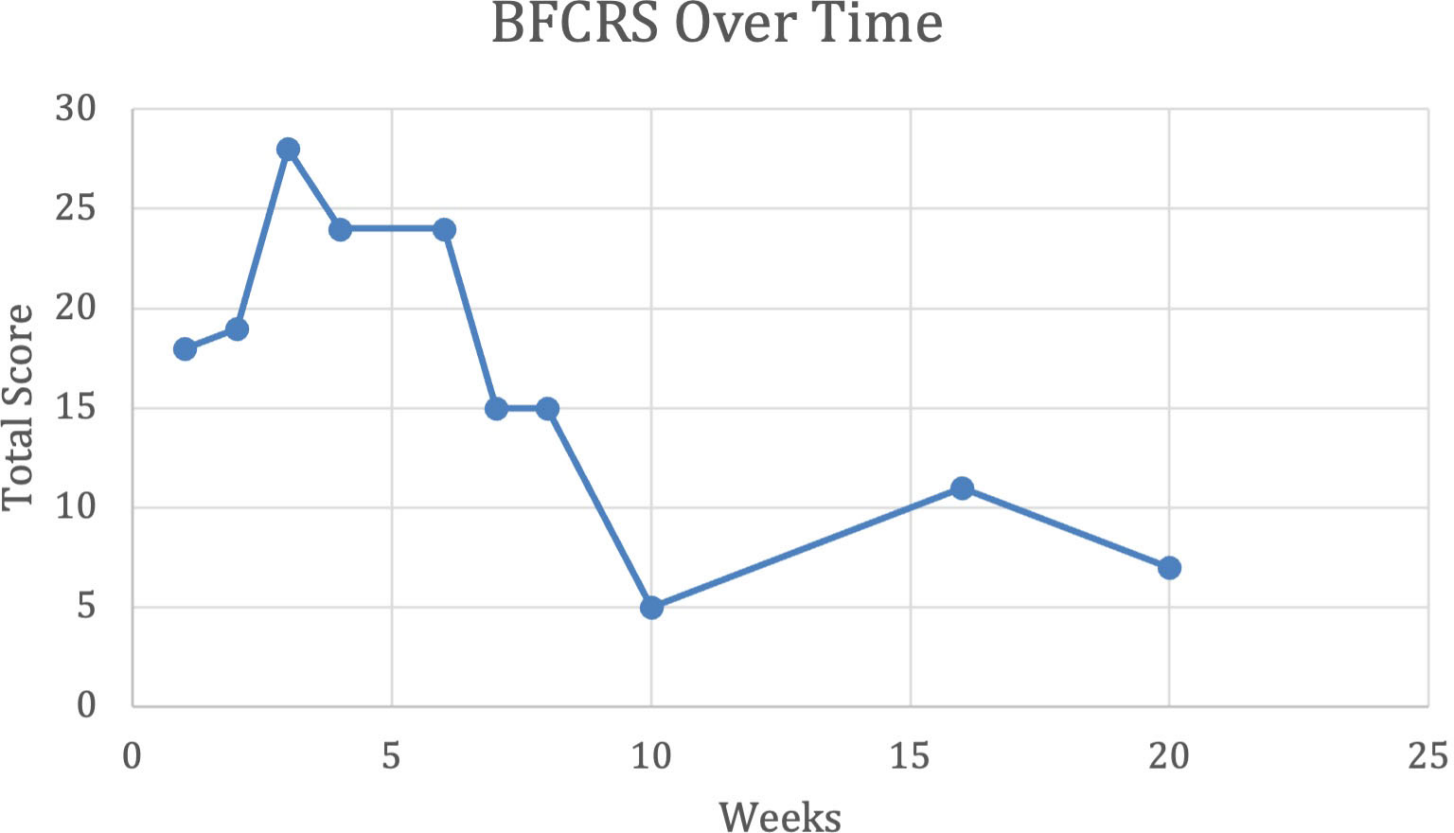
Bush Francis rating score (BFCRS) over time in weeks.

**Figure 3:**
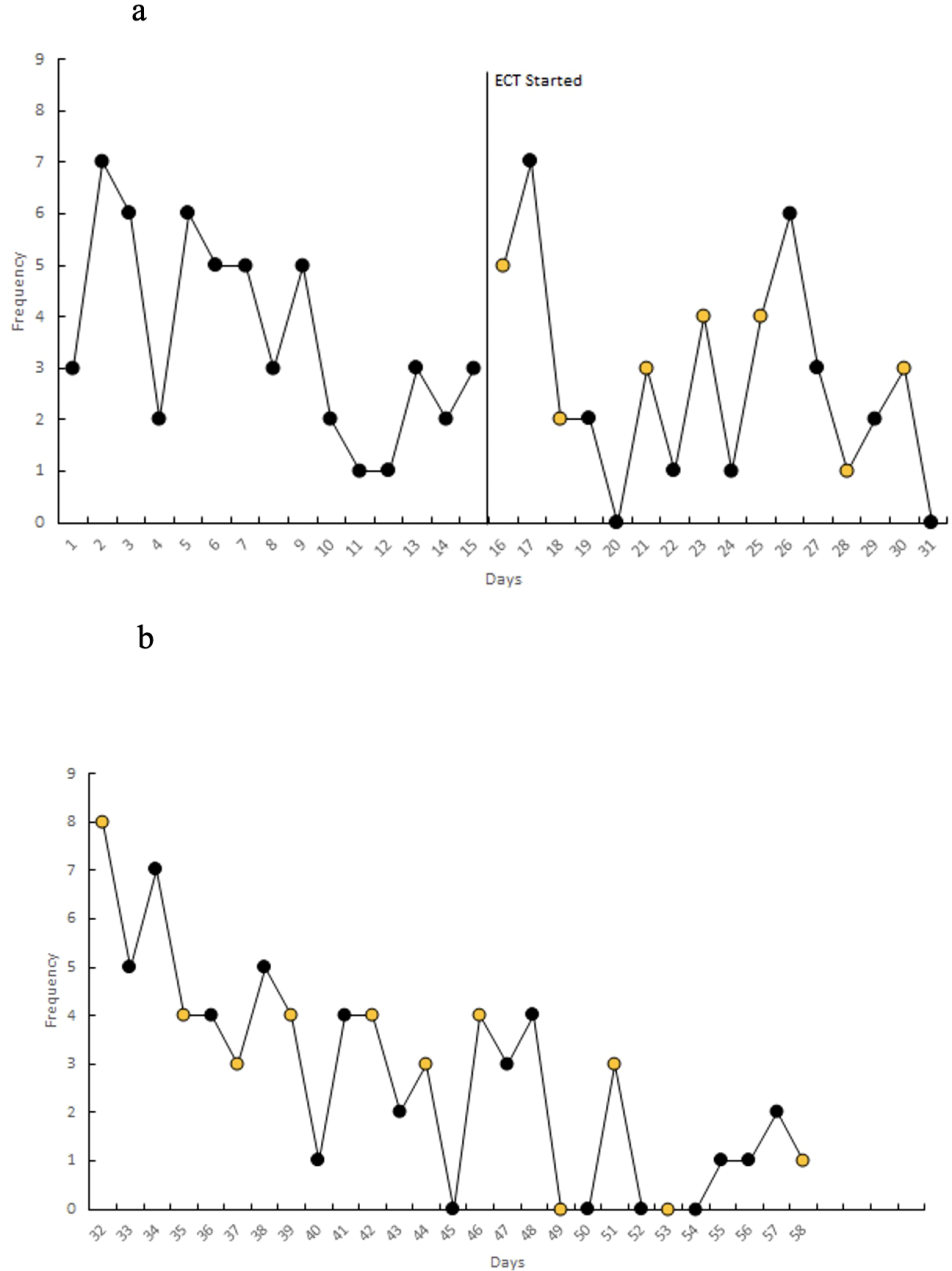
**(A, B)** Daily count of climbing behaviors over time. Key: Yellow dots indicate ECT was received that day.

At the time of discharge, the patient had completed 19 total sessions of ECT (index course of 12 ECT sessions, which was continued by maintenance course with the same three times a week frequency). He was discharged on a slightly reduced clonazepam at 5 mg total daily in divided doses. The BFCRS improved from highest recorded values of 28 to 16. CIS also improved to total frequency 48, total impact 50, and a grand total score of 98. Scores were noticed to further decrease following discharge (refer to **Figure 1** and **2**). Additional improvements noted at discharge were a reduced frequency and severity of the climbing behaviors (refer to **Figure 3**), a decrease in red scale behaviors (such as grabbing, impulsivity), and becoming more redirectable and engaged with the staff. Day time incontinence was still an ongoing concern at discharge, but the patient was able to stay dry for longer periods and could be appropriately toileted more frequently with the assistance. Sleep was stable during the duration of the admission. CGI-I was determined as 2 (much improved) at first follow-up outpatient appointment following discharge.

Standardized rating scales were used to measure change in patient’s symptoms during ECT with baseline score obtained prior to start of ECT. BFCRS was completed regularly by physician and CIS was completed weekly by parents. Additionally, objective data collected by an applied behavior analysis (ABA) therapist, was charted throughout admission. The ABA therapist conducted multiple 10-minute observations for the occurrence of stereotypies. Each of those observations were divided into 10 1-minute intervals and the frequency of behaviors were graphed over time.

We found that the CIS scores showed similar pattern of improvement as identified on BFCRS scores, which can be seen in **Figure 1** and **2** respectively. This data suggests the value of including a parent reported scale (CIS), a clinician rating scale (BFCRS). and objective data gathered by a behavior analyst to help monitor progress during the treatment of catatonia. We noted a slight worsening of symptoms, or plateau in improvement, during the middle of the index course of ECT, which can be observed in **Figure 1** and **2** approximately, after 6 sessions of ECT. This observation was substantiated by both parental and physician report.

## Discussion

*CACNA1A* is a gene on chromosome 19p12 which encodes a subunit of a voltage-gated calcium channel. Studies have suggested that calcium channel genes might be involved in the genetic etiology of ASD. (14, 15). ASD is a neurodevelopmental disorder characterized by deficit in social interaction, impairment in communication skills, and the presence of restricted and repetitive behaviors. Data has shown that there is a higher prevalence of catatonia in those diagnosed with ASD (16, 17). One of the major challenges in diagnosing catatonia in patients with ASD are the many overlapping symptoms between the two conditions. The DSM-5 defines catatonia characterized by the presence of at least three among twelve of the following symptoms: catalepsy, waxy flexibility, stupor, mutism, negativism, agitation, posturing, stereotypes, mannerisms, grimacing, echolalia, and echopraxia. Given the overlap in symptoms between catatonia and ASD, for the purpose of diagnosing catatonia, it is critical to establish a *significant change* from baseline behaviors, or the *emergence of novel* symptoms and/or *identify regression* in individual with ASD (18).

As referenced earlier in the introduction, there is one previously published case report involving an adolescent with ASD and catatonia associated with a *CACNA1A* pathogenic variant who had received ECT for catatonia. He had developed neurologic deficits which were attributed to the ECT (5). Following his first ECT treatment he had an episode of right gaze deviation with right sided clonic activity, which was followed by status epilepticus requiring abortive medication. The patient had right-sided hemiplegia for 72 hours, followed by right-hand weakness that persisted for 1–2 weeks. The patient was discharged to inpatient rehabilitation for ongoing care and made a full neurological recovery. The authors had hypothesized in their publication that patients with psychiatric disorders associated with pathogenic variants in the *CACNA1gene* may be at higher risk for ECT-related complications. The case presented in this paper provides an example where ECT had been administered and effective in an adolescent with a similar genetic disorder with no neurologic complications. A notable difference between the two cases is the use of bilateral ECT in the presented case versus unilateral ECT. The decision was made to use bilateral ECT in this case given the severity of the catatonic illness and lack of response to benzodiazepine treatment, in addition to the difficulty tolerating further titration of the benzodiazepine medication. Most evidence supports the use of bilateral placement given the severity and life threatening risk that presents with catatonia (19) with a treatment frequency at three times a week (10, 20). There are other case reports that have described successful treatment of catatonia with right unilaretal placement, however there are no head to head comparative studies (21).

This case also suggests that longer ECT courses may be beneficial for treatment in this patient population, as the patient had continued to show improvement overtime following ECT index course and through maintenance ECT sessions. At the time of writing this report, the patient had completed a 12-week index course of ECT at a frequency of 3 times a week, and maintenance ECT was continued afterwards at the same frequency for 56 sessions for a total of 68 ECT sessions. Maintenance ECT was still ongoing at the time this paper was submitted. Throughout the ECT course, various pharmacological interventions were used to ensure adequate seizure duration. ECT was administered with no complications observed and progressive improvement was noted in catatonic symptoms. While there is emerging evidence that longer courses of ECT, followed by maintenance ECT may be necessary for the treatment of catatonia, there are no specific guidelines regarding the duration or the frequency of the continuation ECT. Most clinicians treat based on treatment response (22, 23). Fink and Taylor had noted that patients with ASD comorbid with catatonia may need up to 6 months of maintenance ECT to ensure stability with variable treatment frequency determined by the clinician (24).

Another unique lesson learned from this case was that the patient had presented with a previously never-reported symptom of climbing a variety of structures, which had raised serious safety implications. There was discussion surrounding this complex behavior whether it was categorized as a stereotypy, mannerism, driven by perseveration, agitation, impulsivity or related to a mix of symptoms. We ultimately conceptualized that the new onset climbing behavior was a novel motor stereotypy. Mahgoub et al. (25) proposed a new framework for defining stereotypies and mannerisms. In the paper, they suggest stereotypies as non-contextual, fixated repetition of activities and mannerisms as non-contextual, fixated peculiarities of activities (25). The patient’s behaviors were observed to be without purpose and repetitive, and deemed to be more in alignment with the definition of a stereotypy. To date, there is no published literature showing repetitive climbing behaviors in the context of catatonia. This behavior could have been overlooked if the diagnosis was solely reliant symptoms present on a scale, such as the BFCRS. During the hospitalization, climbing behaviors were tracked by a behavior analyst. The patient was observed climbing on the nursing desk, sitting on the basketball hoop in the gymnasium, climbed a swing set, and observed to be sitting atop the door frame of his room. Following ECT, the frequency and severity of climbing behaviors reduced, and the patient became more redirectable from this stereotypy. **Figure 3** shows the reduction in climbing behaviors observed by staff. This positive response to treatment for catatonia further supported our hypothesis that the climbing behavior was possibly a motor symptom of his catatonia.

Regarding the different pharmacological agents used, it is worth commenting on the trial of propranolol for agitation and impulsivity. The patient’s aggressive behaviors were a change from baseline behaviors and coincided with other typically recognized symptoms of catatonia. High-dose propranolol was attempted to target impulsivity and agitation in the outpatient setting, but did not result in any clinical improvement. This is in contrast to a study that identified the efficacy of using high-dose propranolol to target behavior problems in children and adolescents with ASD (26–28). This was not substantiated in the case described here. A notable difference in this case is that behaviors were related to catatonia and not ASD. This may suggest that propranolol is not an effective treatment option to target symptoms of aggression or impulsivity when associated with catatonia, though this is limited by the fact this is a clinical observation in a single case report.

In summary, this case presents an adolescent diagnosed with autism and a *CACNA1A* pathogenic variant who developed catatonia that presented with a unique presentation of a new-onset climbing stereotypy. Catatonia was effectively treated with a prolonged course of bilateral ECT, which was administered with no complications. A strength of this case report is that the treatment response was monitored using multiple standardized instruments, using both physician and parent report, as well as carefully recorded objective data collected by a behavior analyst. This case highlights a few key points. First, patients with rare genetic conditions may be at risk for developing catatonia, and these symptoms may include unusual presentations in this patient population. Second, this case provides support that ECT treatment is effective in this patient population and may require longer treatment. While findings from a single case-report is a limitation and may not be generalizable, use of optimum ECT parameters and pharmacological interventions may provide the best likelihood of a positive response for those who have catatonia associated with *CACNA1* pathogenic variant.

## Data Availability

The original contributions presented in the study are included in the article/**Supplementary Material**. Further inquiries can be directed to the corresponding author.
