# Supplementary material for: Successful bilateral electroconvulsive therapy for catatonia presenting with novel climbing behavior in an adolescent with CACNA1A pathogenic variant and autism spectrum disorder: a case report
Source: Front Psychiatry. 2026 Apr 10;17:1782341. doi: 10.3389/fpsyt.2026.1782341 (PMC13106363; doi:10.3389/fpsyt.2026.1782341)
Supplement: Supplementary file 1 [file Table1.docx]

Ful list of medical work-up: (MRI) brain, Chest X-ray, Electrocardiogram (EKG), Electroencephalogram (EEG ), Complete Blood Count (CBC), Thyroid Stimulating Hormone (TSH) with reflex, Complete Metabolic Panel (CMP), Ammonia, Pediatric Glomerular Filtration Rate (GFR), Creatinine Kinase (CK), Valproic Acid (VPA) level, lumbar puncture (LP), Cerebrospinal Fluid (CSF) autoimmune encephalitis panel, Erythrocyte Sedimentation Rate (ESR), C-Reactive Protein (CRP), Thyroid Peroxidase Antibodies (TPO), Thyrotropin Receptor Antibody, Quantitative Immunoglobins, C3, C4, Antinuclear Antibody (ANA), Lupus Anticoagulant, Beta 2 Glycoprotein, Cardiolipin Antibody, Antineutrophil Cytoplasmic Antibodies (ANCA), Anti-double stranded DNA (Anti-dsDNA), Extractable Nuclear Antibody Panel, von Willebrand antigen, Serum Autoimmune Encephalitis Panel, Urinalysis, Urine Drug Screen (UDS), Gas chromatography–mass spectrometry (GC–MS), and urine porphyrins
